# Low-Cost Mobile Phone Microscopy with a Reversed Mobile Phone Camera Lens

**DOI:** 10.1371/journal.pone.0095330

**Published:** 2014-05-22

**Authors:** Neil A. Switz, Michael V. D'Ambrosio, Daniel A. Fletcher

**Affiliations:** Department of Bioengineering & Biophysics Graduate Group, University of California, Berkeley, California, United States of America; McGill University, Canada

## Abstract

The increasing capabilities and ubiquity of mobile phones and their associated digital cameras offer the possibility of extending low-cost, portable diagnostic microscopy to underserved and low-resource areas. However, mobile phone microscopes created by adding magnifying optics to the phone's camera module have been unable to make use of the full image sensor due to the specialized design of the embedded camera lens, exacerbating the tradeoff between resolution and field of view inherent to optical systems. This tradeoff is acutely felt for diagnostic applications, where the speed and cost of image-based diagnosis is related to the area of the sample that can be viewed at sufficient resolution. Here we present a simple and low-cost approach to mobile phone microscopy that uses a reversed mobile phone camera lens added to an intact mobile phone to enable high quality imaging over a significantly larger field of view than standard microscopy. We demonstrate use of the reversed lens mobile phone microscope to identify red and white blood cells in blood smears and soil-transmitted helminth eggs in stool samples.

## Introduction

The need for disease diagnostics in resource limited settings is well known [1] and has inspired a number of researchers to investigate the possibilities of implementing simple and low-cost microscope systems based around mobile phone cameras [2]–[5]. Such systems leverage the worldwide ubiquity of mobile phone networks together with the image capture and processing power of the phones to create portable devices for disease screening and diagnosis.

Implementation of mobile phone microscopy requires converting a camera designed for conventional photography of meter-scale people into one that can capture diagnostic quality images of micron-scale organisms. To be useful for diagnostic purposes, magnified images must be captured with an appropriate resolution over a sufficiently large sample area to permit a conclusive determination of, for example, whether a cancer cell or infectious agent is present within a large population of normal cells. Resolution and field of view are typically inversely linked in standard laboratory microscopes, with increased resolution due to use of a high-numerical aperture objective with high magnification resulting in a decreased field of view. Large sample areas can nonetheless be obtained by capturing multiple fields of view by manually or automatically moving the sample stage, as is done with commercial slide scanning microscopes used routinely in major hospitals. Mobile phone microscopes are intended for use far from a central hospital, so the field of view and resolution of a single image must both be maximized in order to minimize cost and enable rapid and reliable diagnosis.

Despite the potential of mobile phones to improve access to diagnostic microscopy in low resource settings, current implementations of mobile phone microscopy face significant limitations. The system of Breslauer et al., which adapts a conventional microscope objective to the mobile phone in a portable configuration, creates high-resolution images over a limited field of view that fills only part of the camera sensor and requires several hundred dollars of parts [2]. The mobile phone microscope of Wachsmann, et al., which uses a ball lens for magnification in place of a microscope objective, is lower in cost but creates images with significant aberrations that degrade image quality over the bulk of the field of view [3]. The lens-free holographic approach used by Ozcan, et al. can achieve high resolution over a large field but involves modification of the phone to remove the camera lens, together with computational reconstruction of the image [5]. Furthermore, this approach cannot be used to image samples prepared on standard glass slides due to the requirement that the sample be positioned in close proximity to the sensor. Recently, a low-cost mobile phone microscope based on a ball lens was deployed by Bogoch, et al. in Pemba Island, Tanzania, to screen for helminth infections, but problems with image quality of the ball-lens optics limited the effectiveness of the device [4].

Here we demonstrate that a reversed mobile phone camera lens can be used together with an intact mobile phone camera to capture high quality, wide field images that overcome limitations of previous mobile phone microscope designs. Using a reversed iPhone 4S lens module coupled to an iPhone 4S, we achieved resolution of ≤5 µm over a field of view of ∼10 mm^2^, and useable resolution (≤8 µm) over an area of ∼15.7 mm^2^. Importantly, the camera lenses from mobile phones are low cost (< US$6) due to extremely high manufacturing volumes inherent to mobile phones, enabling construction of a truly low-cost mobile phone microscope that could increase access to diagnostic microscopy in low resource settings.

## Results

**Figure 1:**
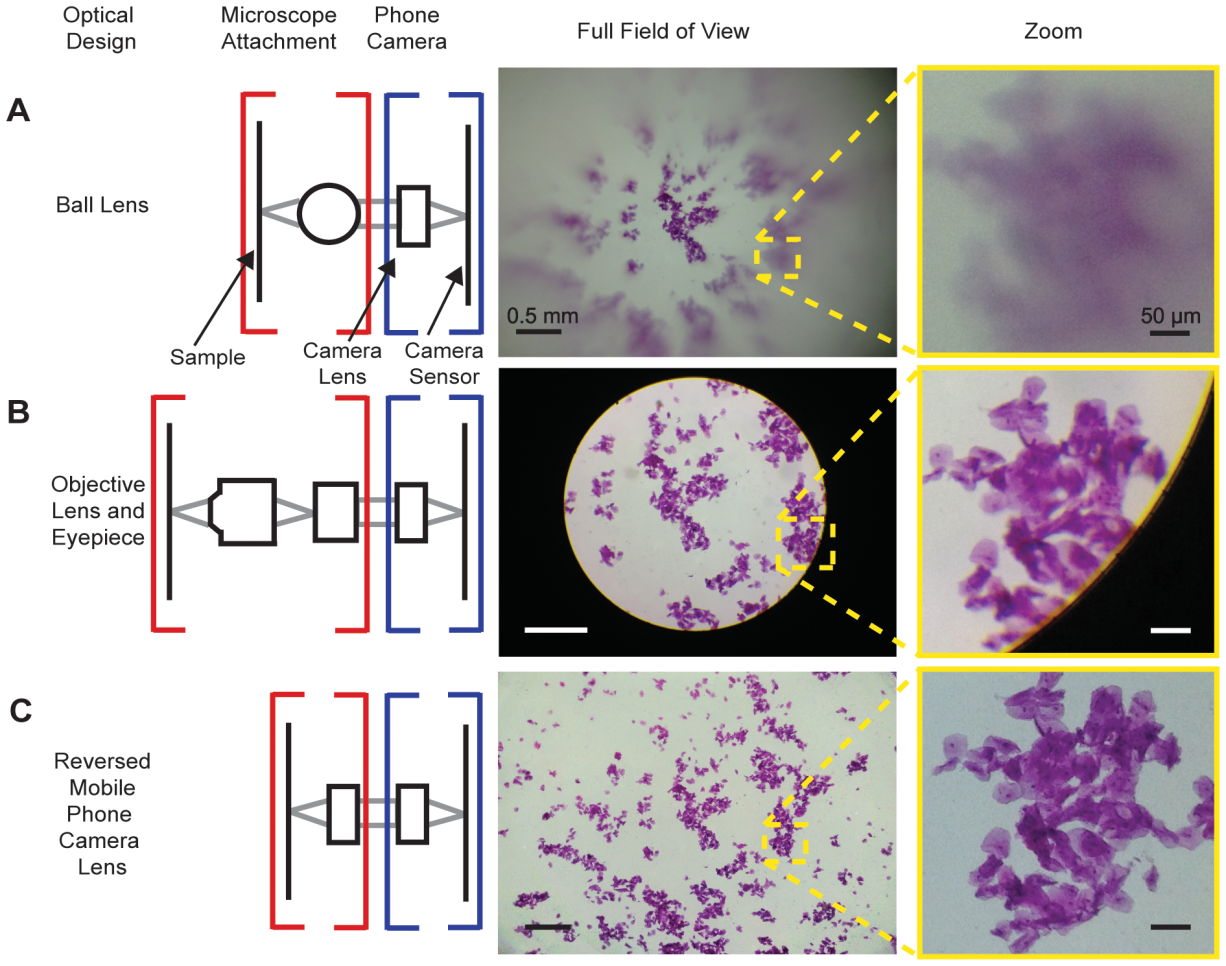
Comparison of mobile phone microscopes. A) *Left panel:* Cartoon schematic of a ball lens mobile phone microscope. Red brackets indicate microscope attachment optics outside of the phone (a ball lens), and blue brackets indicate mobile phone camera optics inside the phone (a lens group and CMOS sensor). *Middle panel:* Image of stained cheek epithelial cells taken with a 6 mm ball lens. *Right panel:* Enlargement of the area indicated within the dashed line in the middle panel. B) *Left panel*: Cartoon schematic of a standard finite objective microscope attachment to a mobile phone, consisting of an objective and an eyepiece. *Middle panel:* Image of stained cheek epithelial cells taken with a 4X/0.10 NA objective and a 20X eyepiece. *Right panel:* Enlargement of the area indicated within the dashed line in the middle panel. Note that despite the image being in-focus at the center of the field of view, some image degradation due to field curvature is detectable at the edge of the field. C) *Left panel:* Cartoon schematic of the reversed lens microscope presented in this paper, with opposing identical lens groups outside the phone (red brackets) and inside the phone (blue brackets). *Middle panel:* Image of stained cheek epithelial cells taken with the opposed lens group setup. *Right panel:* Enlarged area of the area indicated within the dashed line in the middle panel. Note that despite the image being focused at the center of the field, no field curvature is detectable in the reversed lens microscope image, in contrast to the ball lens A) and standard finite objective B) microscope images.

A primary difficulty in doing standard optical microscopy with a mobile phone camera is that achievement of high resolution over a large field of view requires multi-element lens groups to reduce aberrations caused by high collection angles and multiple wavelengths. Such lens groups are typically both expensive and not designed to couple light effectively into the very wide angular field of view – nearly 60° full angle – that mobile phone camera lenses are designed to accept. The need to fill such a wide angular field of view presents limitations for mobile microscopy with either ball lenses or conventional microscope objectives, but this problem can be solved by the use of a reversed mobile phone camera lens as an objective (Figure 1).

The ball-lens (van Leeuwenhoek) style system is a simple approach to mobile microscopy that has a long history (see van Cittert [6], [7], Ford [8], and Steenblik & Steenblik [9]). While having the advantage of requiring only a single ball lens (< US$25), such systems are well known to suffer from a very limited useable field of view (∼50 µm diameter at limiting resolution) due to severe field curvature effects and spherical and other aberrations (Figure 1A). In contrast, a microscope eyepiece is designed to couple light into a wide angular field of view with minimal aberrations, and it can be used in conjunction with a standard objective lens to create a mobile phone microscope [2]. However, microscope eyepieces are designed to receive light from an intermediate image that has a numerical aperture (NA) of approximately (NA_objective_/M_objective_) ∼0.025, where M is the magnification. Consequently, such eyepieces are not well designed for use alone – their low input NA would limit resolution to >12 µm for incoherent 500 nm wavelength (green) light. In addition, such eyepieces are more expensive (> US$60) than ball lenses, and at the lowest price point they provide only a 45° full angular field of view, well below the nearly 60° that phone cameras accept. Consequently, images taken with an eyepiece and objective do not fill the corners of the mobile phone camera sensor (Figure 1B), reducing the field of view from what could have been imaged at that resolution if the full sensor were used.

A lens that is specifically designed to collect light from high angles and well-suited to optically couple to a mobile phone is the mobile phone camera lens itself. Such lenses have f-numbers in the range of 3.0–2.2, equivalent to NAs in the range of 0.17–0.23, which are typical of standard microscope objectives with ∼10× magnification. Conveniently, manufacturing economies of scale have produced extremely inexpensive (<US$6) mobile phone camera lens modules that are better corrected and have a larger number of elements (often twice as many, all with optimized aspheric surfaces) than any moderate-cost eyepieces. A reversed mobile phone camera lens also offers an angular field of view perfectly matched to the camera lens itself, thus making it possible to fully fill the phone image sensor.

To test this idea, we used a reversed iPhone 4S lens (f = 4.28 mm) as an objective in conjunction with a standard iPhone 4S cellphone camera to look at the same stained epithelial cell sample imaged by the ball lens and objective/eyepiece mobile phone microscopes (Figure 1C). Both cell morphology and nuclei are easily discernible across the entire field of view, in contrast to the ball lens objective/eyepiece microscope configurations where field of view and/or resolution are significantly limited (Figure 1A&B). The nominal optical magnification of the image taken with the reversed mobile phone camera lens (Figure 1C) is M = 1, though magnification varies slightly (0.98≤M≤1.06) as sample axial position and thus autofocus vary. As a result, the field of view is nearly identical in size to the sensor (i.e., 4.57×3.43 mm or 15.7 mm^2^). Since the iPhone 4S screen is 3.5-inch diagonal with a 3∶2 aspect ratio, the unzoomed image has an effective magnification M_eff_ = 14.4× when displayed such that the image is not clipped due to the differing aspect ratios of the sensor and screen. Note that because the image sensor has a higher pixel count than the screen – 3264×2448 vs. 960×640 – the image needs to be digitally enlarged on the screen if full resolution information is needed for focusing or diagnosis. For an optical magnification M = 1, sensor pixel pitch of 1.4 µm, and display pixel pitch of 326 ppi, a 1∶1 sensor-to-screen pixel ratio is equivalent to an effective magnification M_eff_ ≈ 56X, though even higher magnification may be preferable for ease in viewing. This tradeoff between M_eff_ and field of view displayed on the phone screen has no impact on the image data captured by the sensor, which is of the full field of view and can be stored for later examination and analysis.

**Figure 2:**
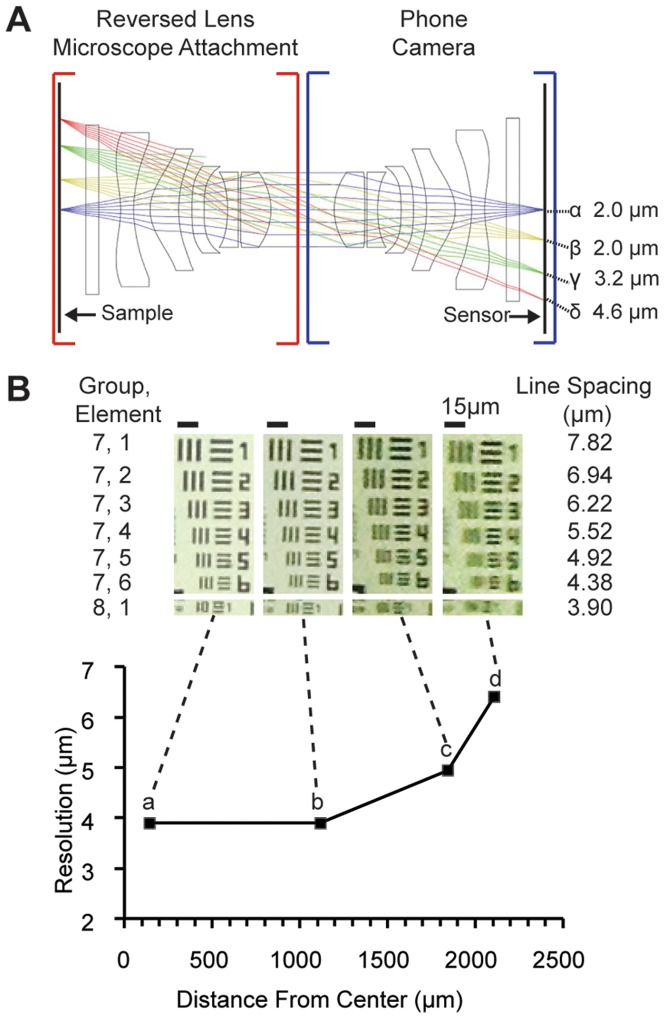
Resolution of the reversed lens microscope. A) Ray-tracing model of a reversed mobile phone camera lens as an objective for a mobile phone microscope. Performance is predicted to be best on axis (α), falling off by >2X at the edge of the field (δ) for a 1.0 mm spacing between lenses. Optical resolution is in microns; to account for variations in sagittal and tangential point-spread at higher field angles, resolution was defined as the first-zero radius of an Airy disk chosen such that its 70% encircled energy radius is the same as that computed for the sample point via ZEMAX. Field positions for α, β, γ, and δ are 0.0, 0.7, 1.5, and 2.1 mm, respectively. B) Measurements of resolution achieved by the reversed lens microscope. The resolution measurements are based on the smallest resolvable group of a 1951 USAF resolution target imaged at different radial distances from the optic axis; asymmetric NA at high field angles (and thus field radii) results in differing sagittal and tangential resolution, as seen in c and d. The dashed line connects to enlargements of the target at the different field positions.

We next evaluated the spatial resolution that could be achieved by mobile phone microscopy with a reversed mobile phone camera lens. In theory, the f/2.4 iPhone 4S camera lens, equivalent to NA 0.21, will have an Airy disk radius (Rayleigh resolution) of ∼1.6 µm at the center of the sensor for light with a wavelength of λ = 550 nm, ignoring any aberrations or sampling issues. To understand the resolution performance in greater detail, we constructed a ZEMAX model of the reversed mobile phone camera lens microscope and compared calculated resolution with measured resolution (Figure 2). The ZEMAX model of the mobile phone camera lens together with its reversed configuration was constructed from a lens design obtained from patent filings by Largan Precision [10], the manufacturer of many of the iPhone lenses. We found that resolution calculated for monochromatic modeling at the patent reference wavelength (Fraunhofer d line, λ = 587.6 nm) varied from 2.0 µm in the middle of the field of view to 4.6 µm at the edge for a lens spacing of 1.0 mm (Figure 2A), with distortion ≲ 0.1% and field curvature predicted to be below 40 µm. This maximum predicted resolution is somewhat worse than the Rayleigh resolution for a perfect lens and may be further reduced in practice by lens manufacturing tolerances, inevitably imperfect chromatic correction, and the inability to perfectly superpose the exit pupil of the reversed lens with the entrance pupil of the cellphone lens.

Digital sampling of magnified images by the pixel array of a camera can also limit the resolution of microscope images. For incoherent illumination, the highest spatial frequency captured from an object is given by k_max_ = 2NA/λ; the pixel pitch for Nyquist sampling must thus be less than or equal to λ/4NA. Substituting this into the Rayleigh resolution formula, δ = 0.61 λ/NA, the sampling limited resolution (ignoring aliasing artifacts) is δ≥2.44 * pixel pitch. The iPhone 4S pixel pitch is 1.4 µm, suggesting a Nyquist-limited resolution (due to the onset of undersampling and aliasing) of ∼3.4 µm for the reversed lens microscope with M = 1. However, assuming a Bayer color filter array, the sampling pitch is actually √2 times the base value in the green (due to the quincuncial arrangement of green pixels) and 2 times the base value in the red or blue, giving 4.8 and 6.8 µm respectively. While the practical resolution limits due to Nyquist sampling will depend on the spectrum of illumination wavelengths, their overlap with transmission bands of the color filters on the sensor, as well as details of the demosaicing algorithms on the phone, these estimates suggest a Nyquist-limited resolution for our system of ∼5 µm. To compare with this estimate, we directly quantified resolution of the reversed lens microscope using images of a 1951 USAF resolution target taken in polychromatic light (see Methods). Our results are quantitatively consistent with Nyquist-limited resolution at the center of the field of view and show decreasing resolution away from the center, as predicted by the ZEMAX model (Figure 2B).

**Figure 3:**
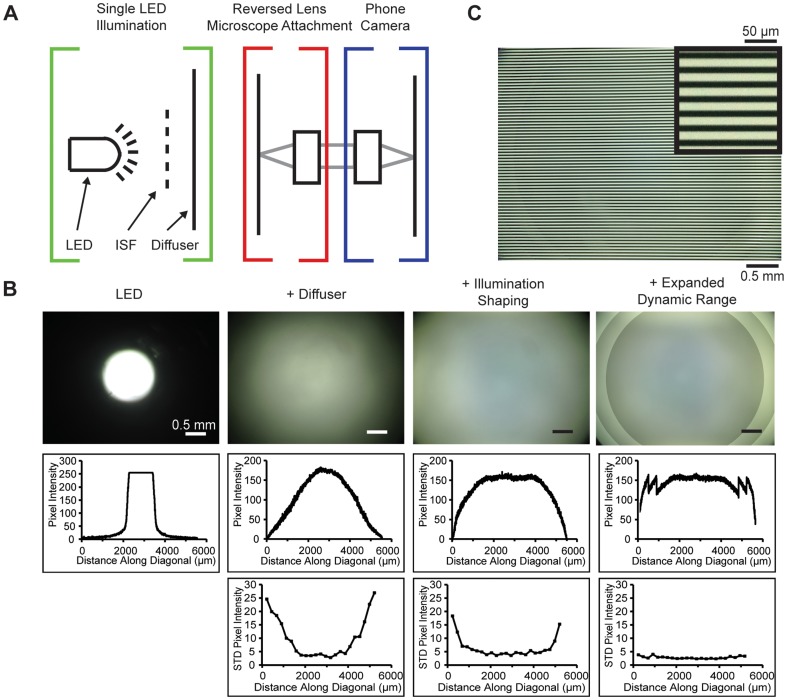
Illumination of the reversed lens microscope. A) Cartoon schematic of the illumination optics together with the collection optics. Red and blue brackets indicate optics outside and inside the phone, respectively. Green brackets indicate the illumination system. A single LED illuminates the sample through an illumination shaping filter (ISF, dashed line) and a diffuser (solid line). B) Methods for correcting image intensity variation caused by vignetting. Columns correspond to the method used. For each column, the top panel is an image of a blank sample showing the illumination uniformity (or lack thereof). The middle panel is a line scan of this image from corner to corner. The lower panel is the standard deviation of a 10×10 pixel box at the indicated positions. Column 1 shows the results of using an LED to directly illuminate the sample. Column 2 shows the results of adding a diffuser between the LED and the sample. Column 3 shows the results of adding illumination shaping filters between the LED and the diffuser. Column 4 shows the results of incorporating a modified form of high-dynamic-range imaging with the diffuser and illumination shaping filters. Images at multiple illumination levels are taken and combined into a single image. Parts of the sample that fall into vignetted regions on the sensor are substituted with the corresponding region of the images taken with brighter illumination levels (see Methods). Note that this image has not yet been flat fielded based on the calibration image. C) An image of a 0.05 mm spacing Ronchi ruling taken with the reversed lens microscope and the combined illumination correction methods described in B). A 10X zoom of a portion of the Ronchi ruling is shown in the upper right corner.

Mobile phone microscopy with a reversed camera lens is subject to significant cos^4th^ vignetting of the image due to collection of light at high field angles from uniformly-illuminated samples [11], potentially limiting the usefulness of the large field of view. Such vignetting has two primary ramifications: first, the variation in maximum intensity across the field can be distracting to the observer and could affect image interpretation; second, changes in contrast across the image restrict the effective dynamic range of the image, which is directly proportional to the degree of vignetting at any given point in the image and will reduce the signal-to-noise ratio (SNR) in different parts of the sample. Vignetting was readily apparent in images taken with our reversed lens microscope when illuminated by a single LED, so we implemented a simple method to correct the effects of vignetting by modifying the illumination profile of our single LED and using high-dynamic-range imaging (Figure 3). This approach produced high quality images across virtually the full sensor (Figure 1C, Figure 3C).

Flat fielding is an alternate approach to illumination correction that removes variation in intensity across an image using a reference frame obtained of a blank sample. However, normalization of maximum intensity in this manner does nothing to remedy the low dynamic range at the sub-optimally-illuminated areas of the image and in fact amplifies image noise in those same areas (as shown in Figure 3B) due to division by low reference image pixel values in precisely the already-vignetted areas. This is especially an issue for mobile phone cameras, where the signal-to-noise is typically low compared to that obtainable with scientific cameras used on standard microscopes.

To counteract this loss of dynamic range and SNR and to reduce the variation in intensity across the image, we modified the illumination profile of the single LED source, which we refer to as illumination shaping, by introducing a mask into the illumination path (Figure 3A). The spatial attenuation profile of the mask was determined by inverting a reference image taken of a blank sample. To maximize simplicity and minimize cost, we printed our illumination shaping filters using transparency stock and a standard laser printer. As a means of quantifying the evenness of the resulting illumination of the sample, we used as a quality metric the number of pixels that were imaged at ≥66% (an arbitrary but large fraction) of maximum brightness (determined as the 99^th^-percentile brightest pixels in the image, to eliminate bias from hot pixels in the low-cost sensors). Illumination with only the single LED and no illumination shaping resulted in only 8.7% of the pixels falling within 66% of maximum brightness (Figure 3B, first column). Including a diffuser made from a piece of wax paper in the illumination path resulted in 34% of the pixels falling within 66% of maximum brightness (Figure 3B, second column). Using illumination shaping with the filters printed on transparency stock together with the diffuser, we were able to bring 81% of the pixels into this range (Figure 3B, third column).

We further improved the uniformity of image intensity and dynamic range by implementing a modified form of high-dynamic-range imaging in addition to illumination shaping. This combined approach produced a composite image in which 95% of the pixels were within 66% of maximum brightness (Figure 3B, fourth column). To generate these composite images, we took three images with increasing illumination brightness. The least-bright image was illuminated for optimal exposure of pixels at the center of the field; the second image for optimal exposure of pixels below 75% of maximum brightness of the first image; and the final image for optimal exposure of pixels near the corners that fell below 75% of the second image. The modulation of illumination level instead of exposure time was necessary because most phones (iOS and Android) allow locking of the current exposure duration but do not allow manual control of exposure settings. Discontinuities along the interface where the images were combined are present but minimal after flat fielding with a composite image taken of a blank sample. Because image noise is amplified by the degree of flat fielding correction required, noise levels (quantified as the standard deviation of pixel intensity) were significantly lowered near the edges of the image as the evenness of the illumination across the field was improved via illumination shaping (Figure 3B). These results demonstrate that substantially uniform illumination with low noise can be achieved with a single LED, diffuser, illumination shaping, and modified high-dynamic-range imaging.

**Figure 4:**
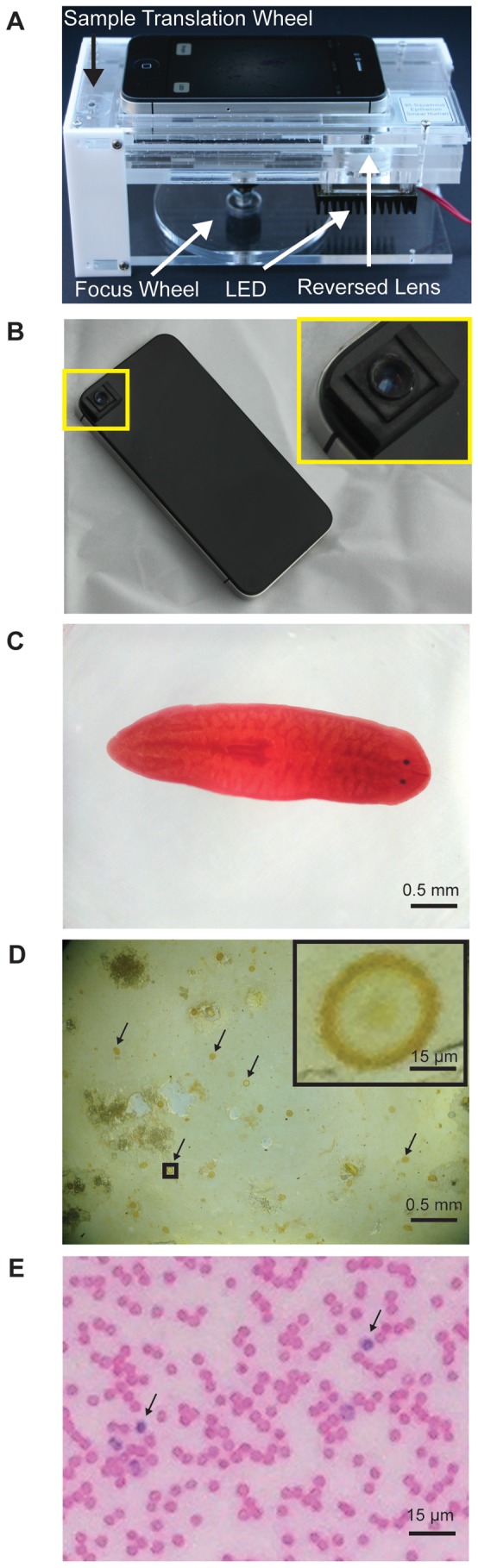
Implementation of the reversed lens microscope. A) Image of the US$30 reversed lens microscope with integrated LED illumination (cost excludes illumination intensity control). This proof-of-concept device features a rack and pinion x- and y-translation mechanism and a screw-based focus mechanism. The reversed mobile phone lens group is fit into a piece of 4.5 mm acrylic, and an LED light source is positioned below the lens. B) Image of a simple lens in a removable lens-mount, all of which can be constructed for nearly the cost of the lens alone. The lens is mounted in acrylic, and slides in an out of tracks that are attached to the phone by a removable adhesive. The contrast of the image area around the camera was enhanced using Photoshop to improve visibility. C) Image of a planarian, taken with illumination shaping. D) Image of a stool sample containing eggs from the soil-transmitted helminth *Ascaris lumbricoides* taken with illumination shaping (sample courtesy of Dr. Isaac Bogoch, Toronto General Hospital). Arrows indicate helminth eggs. Zoomed image of an *Ascaris lumbricoides* helminth egg is shown in the upper right. E) Zoomed image of a blood smear. Arrows indicate white blood cells.

Mobile phone microscopy with a reversed mobile phone camera lens as presented here does not require complicated equipment. Our initial tests of the device used nothing more than the reversed lens fixed to the phone using double-sided tape; centering of the lens was achieved by observing an image and shifting the lens to minimize vignetting. Focus was controlled by pressure against the phone as it lay on a stack of paper adjacent to the sample slide. For convenience, we built an acrylic frame for the phone that incorporated the inverted lens and a low-cost screw-based focusing system and rack-and-pinion XY translation stage costing <US$30 in parts (Figure 4A). As a demonstration of an even cheaper device, we constructed a simple acrylic mount for the lens, adding little to the cost of the lens itself, which was directly coupled to the phone (Figure 4B). While final device designs will depend on the needs of specific applications, we were able to take images of multiple samples such as a planarian, soil-transmitted helminth eggs, and a blood smear mounted on standard microscope slides (Figure 4C, D, E) using the illumination correction approach described above, demonstrating both the large field of view and good resolution of the system.

## Discussion

The use of a reversed mobile phone camera lens as an objective enables high quality mobile phone microscopy with a large field of view at a remarkably low cost. Large fields of view are especially important for point-of-care applications where diagnostic sensitivity is critical. For samples with randomly distributed pathogens (e.g. the blood or sputum smears used for many diagnostics), the probability of getting a false negative – i.e. of measuring n = 0 pathogens in a sample volume V despite the sample containing a true mean of σ pathogens present per unit volume – is given by the Poisson distribution, P(n = 0)  =  e^ – σV^. Due to this exponential dependence on volume interrogated, the benefit of even a slightly larger field of view is substantial. A typical low-cost 5X objective has both a field of view (∼10 mm^2^) and resolution (∼4 µm) similar to that provided by the reversed lens system described above. However, if the slightly degraded resolution (∼6.5 µm) we show at larger field radii remains diagnostically sufficient, then the ≥14 mm^2^ imaged area in the device we describe would allow for an e^1.4^ ∼4X improvement in sensitivity over standard microscopy. Importantly, this system provides microscope-level performance for less than the price of a ball lens. Both the field of view and the Poisson-based sensitivity of the reversed lens microscope are orders of magnitude higher than that of the ball lens microscope with its small (∼50 µm diameter) useable field of view.

The Nyquist sampling limitations that reduce the resolution of iPhone 4S system demonstrated here could be addressed by using inverted mobile phone lenses with different focal lengths. For example, increasing optical magnification by using an iPhone 4S lens (f = 4.28 mm) with a Nokia 808 phone (f = 8.0 mm) could reduce sampling limitations and allow one to make best use of the resolution available from the objective lens, potentially moving closer to theoretically predicted resolutions at the center of the field of ∼2.0 µm, falling to ∼5.0 µm at the image edges. In general, however, the use of production cellphone lenses as objectives inherently limits the range of focal lengths similar to those available in the phones, making it difficult to obtain optical magnifications much beyond M∼2. In cases where larger optical magnification is required for sampling reasons, or resolution beyond ∼2 µm is necessary diagnostically, the use of an objective coupled to a phone via an eyepiece offers more flexibility, though at substantially greater size and cost.

For the reversed lens mobile microscope system we describe here, illumination presents a challenge. The lack of object-side telecentricity when using a mobile phone camera lens as an objective results in substantial cos^4th^ vignetting [11]. Due to the high (∼30°) chief ray angles (CRAs, easily noticeable in the ray bundles emanating from the edge of the sample in Fig. 2A) that mobile phone camera lenses are designed to accommodate, this cos^4th^ factor results in a drop in image intensity by ∼50% at the edges. Beyond this, use of coherent (on-axis) illumination – e.g. from a single LED placed a distance below the sample – is especially problematic as it would result in brightfield imaging in the center of the field, where the acceptance CRA is small, shifting rapidly to darkfield contrast at the periphery of the field where the illumination axis and collection CRA mismatch is significant. Use of a diffuser (Figure 3B, second column) can easily and inexpensively remedy this latter issue of contrast. The vignetting, however, requires additional methods to address, and hence we have used the combined approach of illumination shaping and high-dynamic-range imaging to compensate for it in a simple and compact way. It should be noted that there is room to improve both methods. In a fully realized implementation, the high-dynamic-range illumination modulation technique would automatically calculate both the number of images needed and the spatial assignment of each image to the composite image, instead of using pre-programmed concentric circles. Furthermore, higher contrast printed illumination shaping filters may be able to more completely compensate for the vignetting without the need for additional techniques. The use of LED arrays could provide an alternate route to correcting the vignetting, and resolution could also be improved using the coded illumination approach of Zheng et al [12].

In summary, we have presented and characterized a novel and low-cost mobile phone microscope consisting of a reversed mobile phone camera lens coupled to an intact mobile phone. This system provides low distortion, sub-5 µm resolution over a large field of view for a cost of ∼US$6 beyond the cost of a mobile phone. Furthermore, we have implemented two strategies to control illumination in a manner compatible with mobile phones, and we demonstrated that a US$30 device, in concert with a mobile phone, can deliver high-quality imaging. Due to its simple design and configurability for different applications in the field, this approach to mobile microscopy has the potential to increase the availability and performance of mobile phone microscopes, as well as accelerate their use in resource-limited regions to provide image-based diagnostics.

## Materials and Methods

### Image acquisition

All images, with the exception of those in Figure 1B, were acquired with an iPhone 4S on a custom built acrylic 3-axis translation stage with a phone holder. Either a reversed iPhone 4S lens or a 6 mm ball lens (Edmund Optics Stock #32-746) were fit into this device. The iPhone 4S lens used in this study costs US$15 including the image sensor; similar sample lens groups are available from the manufacturer (Largan Precision) for US$6 [13] and are presumably even less expensive when purchased in quantity. Images in Figure 1B were taken with a 4X 160 mm objective (Edmund Optics Stock #36-131) and a 20X eyepiece (Edmund Optics Stock # 39-696), also using an iPhone 4S. All images were taken with the autofocus fixed to permit the largest field of view. Cheek epithelial cell images are hematoxylin and eosin (HE) stained. The planarian slide and blood smear were purchased from Carolina Biosciences, and the stool sample displaying eggs from the soil-transmitted helminth *Ascaris lumbricoides* was a kind gift of Dr. Isaac Bogoch, Toronto General Hospital.

### Resolution measurements

Resolution was measured using images of a USAF Extreme Resolution Target (Ready Optics). An element was deemed resolvable if the Michelson contrast between the bars was ≥0.1. For noise reduction in the computation, we averaged the image along the bar length, however results are consistent with judgments by eye (c.f. Figure 2B). At higher field angles, resolution in the tangential and sagittal directions varies (c.f. discussion in Figure 2); in these cases we defined a single resolution number as the average between the resolutions along the two axes.

### Illumination Methods

All images, except those in Figure 2, were taken with a single LED and a single diffuser. In Figure 2, an additional frosted-plastic diffuser was placed directly in front of the LED. In order to make the illumination shaping filters, images were taken of an empty field of view. These images were inverted and printed using an HP Color LaserJet printer. To prevent details of the filter from being imaged, the filters were placed directly below the diffuser in the illumination path, approximately 5 mm below the sample. The inclusion of this distance required us to enlarge the filter, which we did in iterative steps until the filter shaped illumination over the entire field of view as desired. Software control of illumination compensation was performed with home-built hardware utilizing a Bluetooth LE linkage between the iPhone and an LED driver controlled by an Arduino. The Bluetooth control, while convenient, does add expense; using the audio jack for control signals would be sufficient in low-cost applications, resulting in a total parts cost for the illumination control system of ∼US$5. All processing was performed on the phone using Objective C, C++, and OpenCV 2.4 (C++ interface). Prior to the image sequence being taken, the phone's exposure was locked at the lowest illumination setting. Three images were then taken at high, medium, and low illumination levels. These three images were combined into a single image in a predetermined manner, using concentric circles to determine which exposure image was used for which area of the final image. The size of the concentric circles and levels of illumination were designed such that the maximum intensities of each sub-region are approximately equal. Pixels within a radius of 1.84 mm of the center of the sensor were taken from the dimmest image, pixels from 1.84 mm to 2.32 mm were taken from the middle intensity image, and pixels from greater than a 2.32 mm radius were taken from the brightest image. Each composite image was then flat-fielded using a similar composite image taken of a blank region of the slide as a reference.
